# Deep Brain Magnetic Stimulation Promotes Neurogenesis and Restores Cholinergic Activity in a Transgenic Mouse Model of Alzheimer’s Disease

**DOI:** 10.3389/fncir.2017.00048

**Published:** 2017-06-30

**Authors:** Junli Zhen, Yanjing Qian, Jian Fu, Ruijun Su, Haiting An, Wei Wang, Yan Zheng, Xiaomin Wang

**Affiliations:** ^1^Department of Neurobiology, Key Laboratory for Neurodegenerative Disorders of the Ministry of Education, Capital Medical UniversityBeijing, China; ^2^Beijing Institute for Brain DisordersBeijing, China; ^3^The Second Hospital of Hebei Medical UniversityShijiazhuang, China

**Keywords:** Alzheimer’s disease, hippocampus, cognition, neurogenesis, cholinergic activity

## Abstract

Alzheimer’s disease (AD) is characterized by progressive decline of memory and cognitive functions. Deep magnetic stimulation (DMS), a noninvasive and nonpharmacological brain stimulation, has been reported to alleviate stress-related cognitive impairment in neuropsychiatric disorders. Our previous study also discovered the preventive effect of DMS on cognitive decline in an AD mouse model. However, the underlying mechanism must be explored further. In this study, we investigated the effect of DMS on spatial learning and memory functions, neurogenesis in the dentate gyrus (DG), as well as expression and activity of the cholinergic system in a transgenic mouse model of AD (5XFAD). Administration of DMS effectively improved performance in spatial learning and memory of 5XFAD mice. Furthermore, neurogenesis in the hippocampal DG of DMS-treated 5XFAD mice was clearly enhanced. In addition, DMS significantly raised the level of acetylcholine and prevented the increase in acetylcholinesterase activity as well as the decrease in acetyltransferase activity in the hippocampus of 5XFAD mice. These findings indicate that DMS may be a promising noninvasive tool for treatment and prevention of AD cognitive impairment by promoting neurogenesis and enhancing cholinergic system function.

## Introduction

Alzheimer’s disease is the most prevalent form of dementia among the elderly and is an emerging global epidemic event (Huang and Mucke, 2012). Although the exact pathogenesis of AD remains elusive, neurotoxicity of amyloid β peptide (Aβ) is considered a major factor in AD pathogenesis (Rajasekhar et al., 2015) and is closely related to impairment of neurogenesis in the AD brain (Zhou et al., 2011).

Neurogenesis, an important mechanism for brain development, is closely associated with hippocampus-mediated learning and memory in the adult DG. These neurons also receive abundant cholinergic innervation, and cholinergic neurotransmitters released from cholinergic neurons are known to play a key role in memory-related circuits in the brain. One such neurotransmitter, Ach, is synthesized from choline and acetyl coenzyme A by ChAT and plays a critical role in learning and memory (Lilja et al., 2015). The duration of Ach action depends on activity of AchE, which hydrolyzes and clears Ach released from the presynaptic membrane. Conversely, removal of this cholinergic innervation through depletion of forebrain Ach impairs neurogenesis in the adult DG (Mohapel et al., 2005). Such deficits in hippocampal neurogenesis have been clearly observed in several animal models of AD (Lilja et al., 2015; Lee et al., 2016). Moreover, a strong association between neurogenesis and degree of cholinergic loss in AD has been demonstrated (Gu et al., 2015).

Since reduced Ach levels were found in the basal forebrain and hippocampus of AD patients and thought to be associated with dementia (Lim et al., 2015), inhibitors of AchE, such as donepezil, rivastigmine, and galantamine, have been used clinically to treat AD patients (Deardorff and Grossberg, 2016). Although several AchE inhibitors have shown neuroprotective effects, multiple side effects limit their utilization in the clinic to treat AD patients. Hence, it is urgent and necessary to explore a safe and effective non-pharmacotherapy intervention for AD.

Deep brain magnetic stimulation, a novel noninvasive approach of brain stimulation, was developed to generate pulses of low-intensity magnetic field (only 1/500 TESLA) by passing a local variable electric current through an inductive coil, which thus could reach the hippocampus directly theoretically due to whole-head penetration (Supplementary Figure S1). It has been found to induce cortical and hippocampal activity and subsequently produce lasting effects (Zhang et al., 2014). Our previous study showed that DMS effectively improved cognitive function in AD mice and significantly decreased amyloid plaque formation in the cortex and hippocampus (Supplementary Figure S2). Furthermore, DMS clearly alleviated stress-related behaviors in animal models by promoting hippocampal neurogenesis (Zhang et al., 2014). However, little is known about the mechanisms through which DMS improves cognitive deficits in AD models.

In the present study, we investigated possible mechanisms underlying DMS -induced stimulation of neurogenesis in the hippocampus and enhancement of cholinergic system activity in 5XFAD mice. To our knowledge, few studies have detected simultaneously behavioral and associated mechanisms of therapeutic effects of DMS treatment in an AD model.

## Materials and Methods

### Animals

APP/PS1 (5XFAD) double transgenic mice coexpress the human APP and PS1 transgenes, which contain 5 familial AD mutations (APPSwFlLon, PSEN1^∗^ M146L ^∗^L286V) under transcriptional control of the neuron-specific mouse *Thy1* promoter. The 5XFAD mice were originally obtained from Jackson Laboratory (number 006554) and maintained by crossing heterozygous transgenic mice with C57BL/6J wild-type breeders. Genotyping was performed using PCR analysis of tail DNA. Female transgenic mice at 4 months of age were utilized, and wild-type littermates served as controls. Mice were housed in cages (4 per cage) in a standard environment (22–25°C, 50–55% relative humidity, and a 12-h light/dark cycle) with free access to food and distilled water. All animal care and use procedures were in strict accordance with the national regulations concerning animal protection and were approved by the animal ethics committee of China Capital Medical University. In sum, all efforts were made to minimize animal suffering and use.

### DMS Treatment

Animals were randomly divided into four groups: wild-type (WT, *n* = 10), WT with DMS (WT + DMS, *n* = 11), 5XFAD (Tg, *n* = 8), and 5XFAD with DMS (Tg + DMS, *n* = 8). The specific parameter settings were based on a previous study (Zhang et al., 2014). Briefly, mice with their cages without metal parts were placed in a DMS machine. Forty-minute successive trains of DMS were administered once daily for 8 weeks. For the WT or Tg groups, conditions were identical to their corresponding DMS group, but they received sham stimulation. As shown the schematic diagram of DMS in the Supplementary Material, the magnetic equipment, including two 360 mm- diameter-coils, was connected to a magnetic field generator and outputs a time- varying magnetic field. The parameter used in this experiment is intermittent Gamma Burst Stimulation (iGBS). Every 2-second-output is composed of several rhythmical trains which spike in intervals of 27, 25, 23, 21, or 19 ms and form the iGBS at 30 ∼ 40 Hz rhythm. The train was composed of 6 pulses with 130 μs width and 1000 Hz frequency. These 2-s runs were separated by an 8-s resting interval, which constitutes the iGBS. In addition, the shape of magnetic fields was changed every 4 min (between linear gradient and approximate distribution), and the rhythm was gradually elevated every 8 min (30, 32.25, 34.5, 37, and 40 Hz).

**FIGURE 1:**
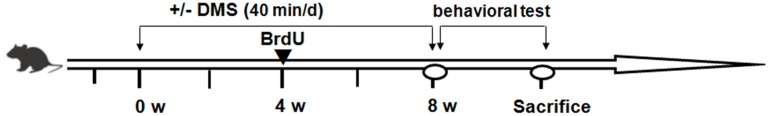
Experimental design and timeline of procedures. Mice in the DMS-treated group were given an 8-week DMS treatment before starting the behavioral tests. During the fourth week before starting behavioral tests, mice received daily BrdU injections (arrowheads).

At the fifth week of treatment, 5-bromo-2-deoxyuridine (BrdU, 50 mg/kg, Sigma–Aldrich, St. Louis, MO, United States) dissolved in physiological saline to make a 10 mg/mL solution was intraperitoneally injected daily for 5 day to label newborn cells in the DG. Behavioral assessments were carried out 24 h after the last DMS treatment. A timeline of experimental procedures is shown in **Figure 1**.

### Morris Water Maze (MWM) Test

Cognitive function was assessed using the MWM test as previously described (Wang Q. et al., 2014). Briefly, the MWM was located in a quiet test room, and the pool was arbitrarily divided into four quadrants. First, a visible platform trial (2 mm above the surface of the water) was performed 24 h prior to experiments to habituate the animal to the experimental environment. Subsequently, the platform was hidden beneath the surface of the water, and each mouse was gently placed in a randomly selected quadrant with its nose pointing toward the wall. Memory acquisition trials were conducted as two sessions daily for five consecutive days; each session comprised 3 trials (starting points were changed for each trial) with an intertrial interval of 30 s and an intersession interval of 2 h. A trial lasted either until the mouse found the platform or for a maximum of 60 s. If a mouse failed to find the platform within 60 s, it was gently guided toward it and left there for 30 s. On the sixth day, the platform was removed for a 60-s probe trial to assess spatial memory retrieval. Number of crossings of the original platform and time spent in the target quadrant (where the platform was located during training) were recorded by a video tracking system and analyzed by Smart software (Actimetrics, Inc.).

### Tissue Preparation

After completion of the behavioral tests, mice were fully anesthetized with 6% chloral hydrate (400 mg/kg, intraperitoneal injection) and perfused transcardially with cold saline solution. Brains were rapidly removed and bisected into each hemisphere through the midsagittal plane. The right hemisphere was fixed in 4% paraformaldehyde in phosphate-buffered saline (PBS) for 48 h and then transferred sequentially to 20% and 30% sucrose solutions in PBS. The brains were sectioned along the coronal plane on a freezing microtome at a thickness of 30 μm in each region (bregma -1.8 mm to -3.24 mm) and stored at -20°C in cryoprotectant solution for staining, including BrdU, BrdU/NeuN, and doublecortin (DCX). The left hemisphere was isolated, and the hippocampus was dissected and stored at -80°C to quantify cholinergic levels, including Ach, ChAT, and AchE.

### BrdU Labeling and Immunofluorescence Staining

BrdU, which is incorporated into DNA during the S-phase, is commonly used as a marker for mitotic cells. Injection of BrdU can be used to evaluate survival time and track the fate of dividing cells and their progeny (Kee et al., 2002). Immunodetection of BrdU and neuronal markers (NeuN) in the DG of the hippocampus was carried out as described previously with some modifications (Wojtowicz and Kee, 2006). Briefly, sections were denatured in 2 N HCl at 37°C for 30 min, incubated in blocking solution (10% goat serum, 0.3% Triton X-100 in 0.01% PBS) for 1 h, and then incubated with rabbit anti-BrdU (1:200, ab6326, Abcam) and mouse monoclonal anti-NeuN (1:100, MAB377, Millipore, MA, United States) for 48 h at 4°C. After rinses with PBS, slices were immersed in biotin-SP-conjugated goat anti-rabbit IgG Alexa Fluor 488 (1:200, A-11034, Invitrogen) and anti-mouse Alexa-Fluor 594 dyes (1:250, A-11032, Invitrogen) for 2 h at room temperature. Slices were then mounted on gelatin-coated slides.

We used the stereological techniques to count the same one side, the number and phenotype of BrdU^+^ cells were analyzed as described previously (Wang et al., 2015). Briefly, confocal and z-stacked images were used to determine the number of BrdU^+^ cells colabeled with NeuN (BrdU^+^/NeuN^+^ cells). The number of BrdU^+^ cells or BrdU^+^/NeuN^+^ cells in every sixth section (180 mm apart) was obtained from eight sections, multiplied by 6, and then multiplied by 2 to account for both hemispheres.

### DCX Immunohistochemical Staining

DCX, a microtubule-stabilizing factor expressed early in neuronal differentiation, is a reliable marker of newly migrating neuroblasts in the adult DG (Verret et al., 2007). Briefly, a 1:6 series of sections were stained, and mounted sections were defatted in xylene and hydrated in a series of ethyl alcohols and water. Sections were incubated in primary rabbit polyclonal DCX antibody (1:200, Cell Signaling Technology Inc., Beverly, MA, United States) for 1 h at room temperature and then overnight at 4°C. After washing thoroughly, slices were reacted with goat anti-rabbit IgG (1:200, Vector Laboratories). Subsequently, chromogen development with DAB (200 mg/mL TB, 0.01% H_2_O_2_) was carried out for 30 min. Quantification of DCX^+^ cells in sections of each group was carried out using Image Pro Plus 6.0 software.

### Assay of Ach, ChAT, and AchE

Cholinergic markers were measured as described previously study (Ruan et al., 2010). Briefly, the hippocampal supernatant was further diluted with a buffer solution appropriate for the relevant biochemical index. The level of Ach in the supernatant was detected with an ELISA kit (A105-1, Nanjing Jiancheng Bioengineering Institute, China) according to the manufacturer’s instructions. Each reaction contained a 100 μL sample, 0.5 U/mL AchE, 200 μM Amplex Red reagent, 0.1 U/mL choline oxidase, and 1 U/mL HRP in reaction buffer. The reaction systems were incubated at room temperature for 30 min with protection from light, and then fluorescence intensity was measured at an excitation wavelength of 560 nm and an emission wavelength of 580 nm. Similarly, the activities of ChAT and AchE were detected spectrophotometrically with commercial assay kits (A079-1 and A024, Nanjing Jiancheng Bioengineering Institute) and are described in units per milligram fresh tissue protein.

### Statistical Analysis

All values are shown as mean ± standard error of mean (SEM). All data were performed by an investigator who was blinded to the experimental conditions. The following tests were used as required: Data recorded in the training trail (escape latency and swimming speed) were analyzed by two-way analysis of variance (ANOVA) with repeated measures using SPSS 13.0 software (SPSS, Seattle, WA, United States), followed by LSD *post hoc* test to compare differences between groups, and *p* < 0.05 was considered statistically significant. Other data (when comparing three or more groups) were analyzed by one-way ANOVA followed by Bonferroni *post hoc* test using Prism 5.0 (GraphPad Software).

## Results

### DMS Rescues Spatial Cognitive Impairment in 5XFAD Mice

**FIGURE 2:**
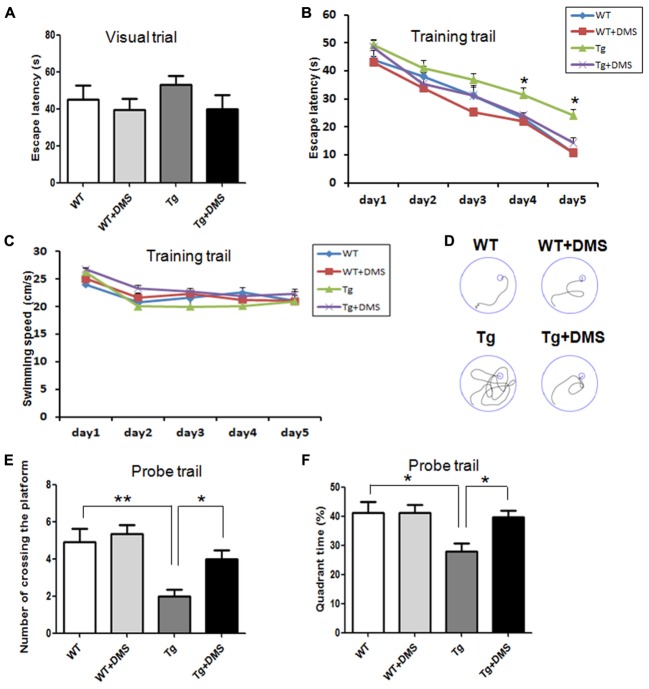
Deep magnetic stimulation treatment improved spatial learning and memory of 5XFAD mice in the Morris water maze (MWM). Escape strategies during the visible platform task adopted by WT, WT + DMS, Tg, and Tg + DMS groups were detected using a camera and included escape latency **(A)**. The escape latency **(B)** and average swimming speed **(C)** of mice to find the hidden platform were recorded on each training day. **(D)** One day after finishing the acquisition task, a probe trial was performed to evaluate spatial memory. Four representative swim traces are shown for each group. Number of times crossing the platform **(E)** and the quadrant time crossing over the platform **(F)** were used to determine memory retention of these groups of mice. All values represent mean ± SEM. ^∗^*p* < 0.05 and ^∗∗^*p* < 0.01 vs. Tg; WT (*n* = 10), WT+DMS (*n* = 11), Tg (*n* = 8), Tg+DMS (*n* = 8).

To determine whether DMS alleviates Aβ-induced cognitive impairment, we tested spatial learning and memory ability of 5XFAD mice in the MWM following DMS treatments. In the visible platform test, there was no group difference in swimming strategies (*p* = 0.538, **Figure 2A**). Subsequently, in the acquisition phase, mice from all groups showed a progressive decline in escape latency during training (**Figure 2B**), and this decline was more significant as the training days progressed in the DMS treatment group. Mice in the Tg group exhibited prolonged escape latencies during all sessions compared with the control mice, while mice in the Tg + DMS group significantly decreased in escape latency compared with the Tg group from the fourth day (*p* < 0.05). However, the average swimming speed did not differ significantly (*p* = 0.41, **Figure 2C**) among the groups in this trial, which indicated that DMS treatment substantially improved learning deficits. In addition, to evaluate spatial memory retention, a probe trial was performed **Figure 2D**. Similarly, mice in the Tg + DMS group crossed the former platform location more often and spent more time in the target quadrant than those in the Tg group (*p* < 0.05, **Figures 2E,F**), suggesting that DMS effectively reversed spatial memory impairment in AD mice.

### DMS Enhances Adult Hippocampal Neurogenesis in 5XFAD Mice

**FIGURE 3:**
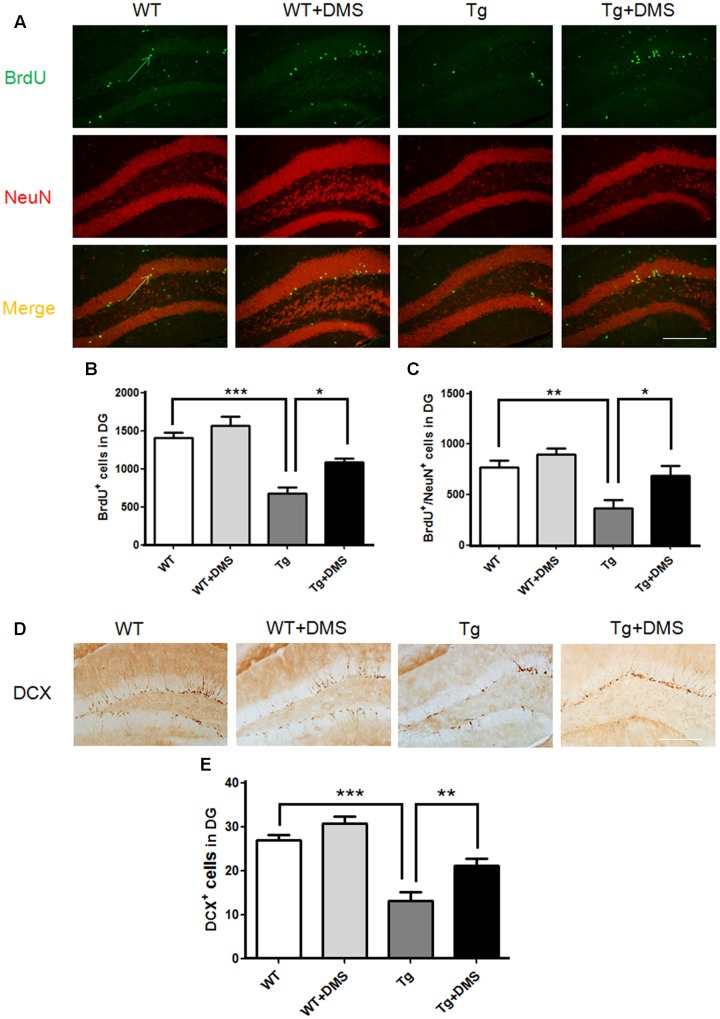
Deep magnetic stimulation treatment promoted neurogenesis in the DG in 5XFAD mice. **(A)** Representative fluorescence images of confocal [BrdU^+^ cells (green), NeuN^+^ cells (red), BrdU^+^/NeuN^+^ (merge)] in the DG of each group of mice. The total number of BrdU^+^ cells **(B)** and newly generated neurons **(C)** in the DG increased after DMS treatment compared with AD mice without DMS treatment. All micrographs are shown at 200× magnification. Scale bar = 200 μm. Data are expressed as mean ± SEM, ^∗^*p* < 0.05, ^∗∗^*p* < 0.01, and ^∗∗∗^*p* < 0.001 vs. Tg. **(D)** Representative images of DCX^+^ cells in the granule cell layer of each group of mice, in agreement with the location of neural precursor cells of the DG. Scale bar = 200 μm. **(E)** DMS treatment increased the number of DCX^+^ cells in the DG of 5XFAD mice. Data are expressed as mean ± SEM. ^∗∗^*p* < 0.01, ^∗∗∗^*p* < 0.001 vs. Tg. *n* = 8/group.

The thymidine analog BrdU was injected to label dividing neural progenitor cells. After 8 weeks of DMS treatment, BrdU^+^ cells were counted in the DG region to determine survival of cells, and a significant group difference was found (*p* < 0.001, **Figure 3A**). Compared with the WT group, mice in the Tg group showed not only a decreased number of surviving BrdU^+^ cells (672.00 ± 85.87 vs. 1408.00 ± 68.63, *p* < 0.001, **Figure 3B**) but also a decreased number of new neurons (BrdU^+^/NeuN^+^ cells, yellow) in the DG (368.00 ± 76.06 vs. 768.00 ± 70.11, *p* < 0.01, **Figure 3C**). However, marked increases in the number of BrdU^+^ cells (1088.00 ± 53.55 vs. 672.00 ± 85.87, *p* < 0.05) and BrdU^+^/NeuN^+^ cells (688.00 ± 97.32 vs. 368.00 ± 76.06, *p* < 0.05) were observed in the Tg + DMS group compared with the Tg group, suggesting that DMS could promote differentiation of newborn cells into mature neurons.

In addition, the number of DCX^+^ cells in the DG was markedly reduced in the Tg group compared with the WT group (12.28 ± 1.70 vs. 26.85 ± 1.33, *p* < 0.001, **Figures 3D,E**), while DMS treatment dramatically increased the number of DCX^+^ cells compared with that of the Tg group (21.04 ± 1.71 vs. 12.28 ± 1.70, *p* < 0.01), indicating that DMS treatment effectively promoted neurogenesis in the hippocampus of AD mice.

### DMS Restores Decreased Ach Levels and ChAT Activity and Increases AchE Activity in the Hippocampus of 5XFAD mice

**FIGURE 4:**
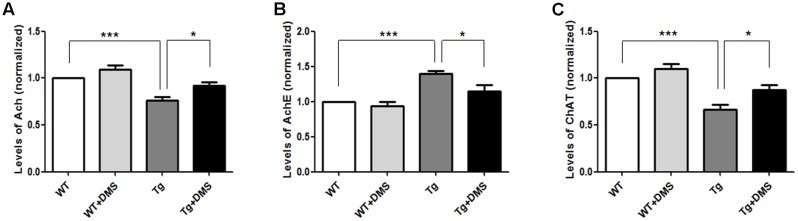
Effect of DMS on the levels of Ach **(A)**, AchE **(B)**, and ChAT **(C)** in the hippocampal homogenate. DMS treatment restored decreased Ach levels and ChAT activity and increased AchE activity in 5XFAD mice. Values are expressed as mean ± SEM. ^∗^*p* < 0.05, and ^∗∗∗^*p* < 0.001 vs. Tg. *n* = 8/group.

To determine whether DMS could regulate the activity of cholinergic system, we tested some cholinergic associated factors in the hippocampus of 5XFAD mice following DMS treatments. As shown in **Figures 4A,C**, compared with the WT group, the content of Ach (75.93 ± 3.80% vs. 100%, *p* < 0.001, **Figure 4A**) and ChAT (66.43 ± 5.53% vs. 100%, *p* < 0.001, **Figure 4C**) significantly decreased to about 24.07 and 33.57%, respectively, and the activity of AchE markedly increased in the Tg group (140.10 ± 3.80% vs. 100%, *p* < 0.001, **Figure 4B**). However, compared with the Tg group, mice in the Tg + DMS group showed partial restoration of Ach levels (91.67 ± 3.97% vs. 75.93 ± 3.80%, *p* < 0.05) and ChAT activity (87.85 ± 4.85% vs. 66.43 ± 5.53%, *p* < 0.05), as well as inhibition of AchE activity (115.10 ± 8.20% vs. 140.10 ± 3.80%, *p* < 0.05), demonstrating that DMS treatment effectively regulated cholinergic system balance in AD mice.

## Discussion

In the current study, we verified therapeutic effects of DMS, a novel and promising therapeutic approach, on cognitive impairment in an AD mouse model and investigated underlying mechanisms. Consistent with our previous observations, DMS effectively rescued learning and memory in 5XFAD mice. Furthermore, DMS clearly promoted neurogenesis and restored cholinergic system balance in the hippocampus of 5XFAD mice, both of which are critical to hippocampal learning and memory.

Several researchers have reported that 5XFAD double transgenic mice showed significant cognitive decline at 4–5 months of age (Oakley et al., 2006; Wang Q. et al., 2014). Once interventions for halting learning and memory deficits are available, they are likely to be more beneficial during early phases of AD. Therefore, we used 4-month-old transgenic mice and their littermates to perform the study. As expected, DMS treatment markedly prevented behavioral impairments not only in the training trial but also in the probe trial in 5XFAD mice. Our results reinforce the conclusion that DMS treatment effectively improves hippocampus-dependent spatial learning and memory ability in AD mice. Since the relevant mechanisms underlying effects of DMS on cognitive function in AD mice are not known, we aimed to elucidate these mechanisms.

Overwhelming evidence shows that the adult brain of humans and other mammals continuously generates new neurons throughout life (van Praag et al., 2002; Villeda et al., 2011), which involves regulation of multiple physiological steps including cell proliferation, migration, and subsequent differentiation of cells into mature neurons. However, neurogenic capacity declines with age and neurodegenerative diseases such as AD (Perry et al., 2012; Jeong et al., 2014; Tanaka et al., 2015). Given that DMS has been shown to promote hippocampal neurogenesis in stress-induced animal models (Zhang et al., 2014), we performed BrdU injection for five consecutive days to determine whether DMS treatment also promoted hippocampal neurogenesis in AD transgenic mice. Similar to previous research findings (Moon et al., 2014; Zeng et al., 2016), we found that 5XFAD mice demonstrated reduced differentiation into granule neurons and incorporation into adult hippocampal circuitry, which may contribute to cognitive deficits. DMS administration significantly increased the number of newborn neurons and immature neurons identified as BrdU^+^/NeuN^+^ cells as well as the number of DCX^+^ cells in the DG of 5XFAD mice, suggesting that deficits in proliferation and differentiation of neural progenitor cells in AD transgenic mice could be rescued by DMS. Thus, DMS may improve cognitive function at least in part by promoting neurogenesis in the hippocampus.

How does DMS treatment promote hippocampal neurogenesis in 5XFAD mice? According to previous studies, the cholinergic system plays a pivotal role in promoting survival of neuronal progenitors in adult neurogenesis. Moreover, Aβ oligomers are considered a primary cause of impairment of the cholinergic system in the brains of AD patients, which show multiple aspects of cholinergic system dysfunction such as decreases in Ach levels, low activity of choline synthetase, and a reduction in the number of cholinergic receptors (Bao et al., 2012; Yang et al., 2014). In addition, studies have reported that suppression of cholinergic signaling readily exerts a detrimental effect on neurogenesis in the hippocampus of AD models (Perry et al., 2012; Tanaka et al., 2015); however, an effective intervention method for AD in the clinic has not yet been found. Thus, we examined whether DMS exerts its effects by enhancing cholinergic activity in the hippocampus of AD mice. In our study, actions of cholinergic neurotransmitters were clearly decreased in the hippocampus of 5XFAD mice, while DMS treatment effectively restored this activity. Thus, regulating cholinergic activity and promoting neurogenesis in the hippocampus may be pivotal mechanisms through which DMS improves cognitive function in AD mice. To our knowledge, DMS has never been used to examine simultaneously cholinergic activity and cognitive benefits in AD. However, due to complex pathological mechanisms of AD, further work will explore additional DMS targets in AD, such as synaptic plasticity and oxidative stress. The molecular mechanisms of DMS-mediated neuroprotection are just beginning to be explored.

Furthermore, several researches reported the prospects of modulating Wnt signaling as a strategy for neuroprotection (Inestrosa and Varela-Nallar, 2014). This will include the potential of Wnts to: (i) act as potent regulators of hippocampal synapses and impact in learning and memory; (ii) regulate adult neurogenesis; and finally (iii) control AD pathogenesis. Also, inducing adult neurogenesis through activation of the canonical Wnt/β-catenin pathway and may offer a therapeutic approach to treating AD, by enhancing a brain self-repair mechanism (Tiwari et al., 2014). Previous work shows that magnetic stimulation can directly regulate neuronal circuits and improve brain functions (Wang J. X. et al., 2014). Hence, we suspect that whether DMS also could regulate the neurogenesis through activation of the canonical Wnt/β-catenin pathway and regulate neural network activity to hinder AD progression in the follow-up study, which might be a novel mechanism involving in DMS treatment.

The limitations to our study are that we have not performed dynamic observations of DMS effects on hippocampal neurogenesis and cholinergic systems in AD mice, and we have not explored critical signals regulating neurogenesis (Hollands et al., 2016).

## Conclusion

Our results suggest that DMS may effectively improve learning and memory performance in 5XFAD mice by restoring cholinergic activity and promoting hippocampal neurogenesis. Furthermore, our findings indicate that DMS could be a new noninvasive therapeutic strategy for AD and other aging-associated cognitive impairments. In the further study, we will expore the probable molecular mechanism of DMS in regulating the neurogenesis.

## Ethics Statement

This study was carried out in accordance with the recommendations of National Institutes of Health guidelines for care and use of laboratory animals and with the European Communities Council Directive of 24 November 1986 (86/609/EEC).

## Author Contributions

JZ and XW contributed to the design, acquisition of the data, and the draft of the manuscript. YQ, JF, HA, and WW performed the whole experiments and helped acquire and interpret of the data. RS contributed to analyze the data and discuss the results. YZ and XW provided intellectual contribution during editing of the manuscript.

## Conflict of Interest Statement

The authors declare that the research was conducted in the absence of any commercial or financial relationships that could be construed as a potential conflict of interest.

## Abbreviations

5XFAD: a transgenic mouse model of AD
Aβ: amyloid β peptide
Ach: acetylcholine
AchE: acetylcholinesterase
AD: Alzheimer’s disease
ChAT: choline acetyltransferase
DG: dentate gyrus
DMS: Deep magnetic stimulation
