# Supplementary material for: Deep Brain Magnetic Stimulation Promotes Neurogenesis and Restores Cholinergic Activity in a Transgenic Mouse Model of Alzheimer’s Disease
Source: Front Neural Circuits. 2017 Jun 30;11:48. doi: 10.3389/fncir.2017.00048 (PMC5492391; doi:10.3389/fncir.2017.00048)
Supplement: Supplementary file 1 [file Presentation_1.pdf]

Supplementary Material

Deep Brain Magnetic Stimulation Promotes Neurogenesis and Restores Cholinergic Activity in a Transgenic Mouse Model of Alzheimer's Disease

Junli Zhen[1,2,3], Yanjing Qian[1,2], Jian Fu[3], Ruijun Su[1,2], Haiting An[1,2], Wei Wang[1,2], Yan Zheng[1,2,*], Xiaomin Wang[1,2,*]

[1]*Department of Physiology, Department of Neurobiology, Key Laboratory for Neurodegenerative Disorders of the Ministry of Education, Capital Medical University, Beijing 100069, PR China.*
[2]*Beijing Institute for Brain Disorders,Beijing 100069, PR China.*
[3]*The Second Hospital of Hebei Medical University,Shijiazhuang 050000, PR China.*

***Authors for correspondence** *(zhengyan@ccmu.edu.cn; xmwang@ccmu.edu.cn)*

1 Supplementary Materials and Methods

1.1 Equipment

As shown in the schematic diagram of DMS (**Figure S1**), the parameter used in this experiment is intermittent Gamma Burst Stimulation (iGBS).

1.2 Immunostaining

Brains were cryoprotected in 30% sucrose solution in 0.01 M PBS for 24 h at 4℃and then sectioned on a freezing microtome (Model:2165; Leica, Bensheim, Germany) at 30 μm. A standard avidin-biotin complex (ABC) staining method was utilized to assess the distribution and morphology of amyloid plaques. The mouse monoclonal anti-Aβ antibody (6E10, 1:1000; SIG-39300, Covance) and the secondary antibody, biotinylated goat anti-mouse IgG (1:200, Vector Laboratories), were used. The sections were air-dried, dehydrated with serial concentrations of ethanol (70%, 80%, 90% and 100%), hyalinized in xylene and coverslipped. The images were observed under a light microscope (Olympus, **Figure S2**) and the areas of Aβ-positive plaques were quantified using Image Pro Plus 6.0 software according to our previous description (Zheng et al., 2012). Ten consecutive sections of each brain were imaged together, and the areas occupied as well as average size by Aβ-positive staining in each section were measured. The mean value calculated across images from each brain was considered one sample (n = 6/group).

1.3 Behavioral Assessment

Novel object recognition test was performed based on a previous protocol (Bevins and Besheer, 2006). Briefly, mice were placed in an experimental environment without any object for 5 min at the first day. Next day, mice were trained to explore two identical objects (object A) for 5 min. After 24 h, one familiar object A was replaced by a novel object (object B). Mice were allowed to explore objects A and B for 5 min (**Figure S3**). The exploration time for the object A (TA) and the object B (TB) was recorded in blind trails. The number of novel object contacting and novel object recognition index (TB/[TA + TB]) was calculated.

1.4 Statistical Analysis

All data are shown as mean ± standard error of mean (SEM). The data were analyzed using one-way ANOVA followed by Bonferroni's *post hoc* test for comparisons among more than two groups or Student's *t*-test for two-group comparisons. All statistical analysis was performed with SPSS 13.0 software. $p < 0.05$ was considered statistical significance.

1.5 REFERENCES

Bevins RA, Besheer J (2006). Object recognition in rats and mice: a one-trial nonmatching-to-sample learning task to study 'recognition memory'. *Nat Protoc* 1:1306-1311. doi:10.1038/nprot.2006.205

Zheng Y, Wang Q, Xiao B, Lu Q, Wang Y, Wang X (2012). Involvement of receptor tyrosine kinase Tyro3 in amyloidogenic APP processing and beta-amyloid deposition in Alzheimer's disease models. *PLoS One* 7:e39035. doi: 10.1371/ journal.pone.0039035

2 Supplementary Figures

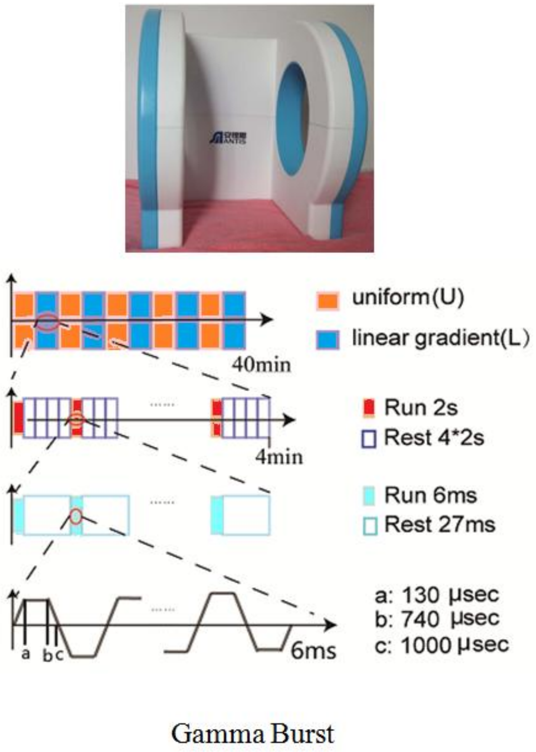

FIGURE S1 | The schematic diagram of DMS (It was designed and made by Beijing Aldans Biotech Co., Ltd)**.** The parameter used in this experiment is intermittent Gamma Burst Stimulation (iGBS). Every 2-second-output is composed of several rhythmical trains which spike in intervals of 27, 25, 23, 21, or 19 ms and form the iGBS at 30 ~ 40 Hz rhythm. The train was composed of 6 pulses with 130 µs width and 1000 Hz frequency. These 2-second runs were separated by an 8-second resting interval, which constitutes the iGBS. In addition, the shape of magnetic fields was changed every 4 min (between linear gradient and approximate distribution), and the rhythm was gradually elevated every 8 min (30, 32.25, 34.5, 37, and 40 Hz).

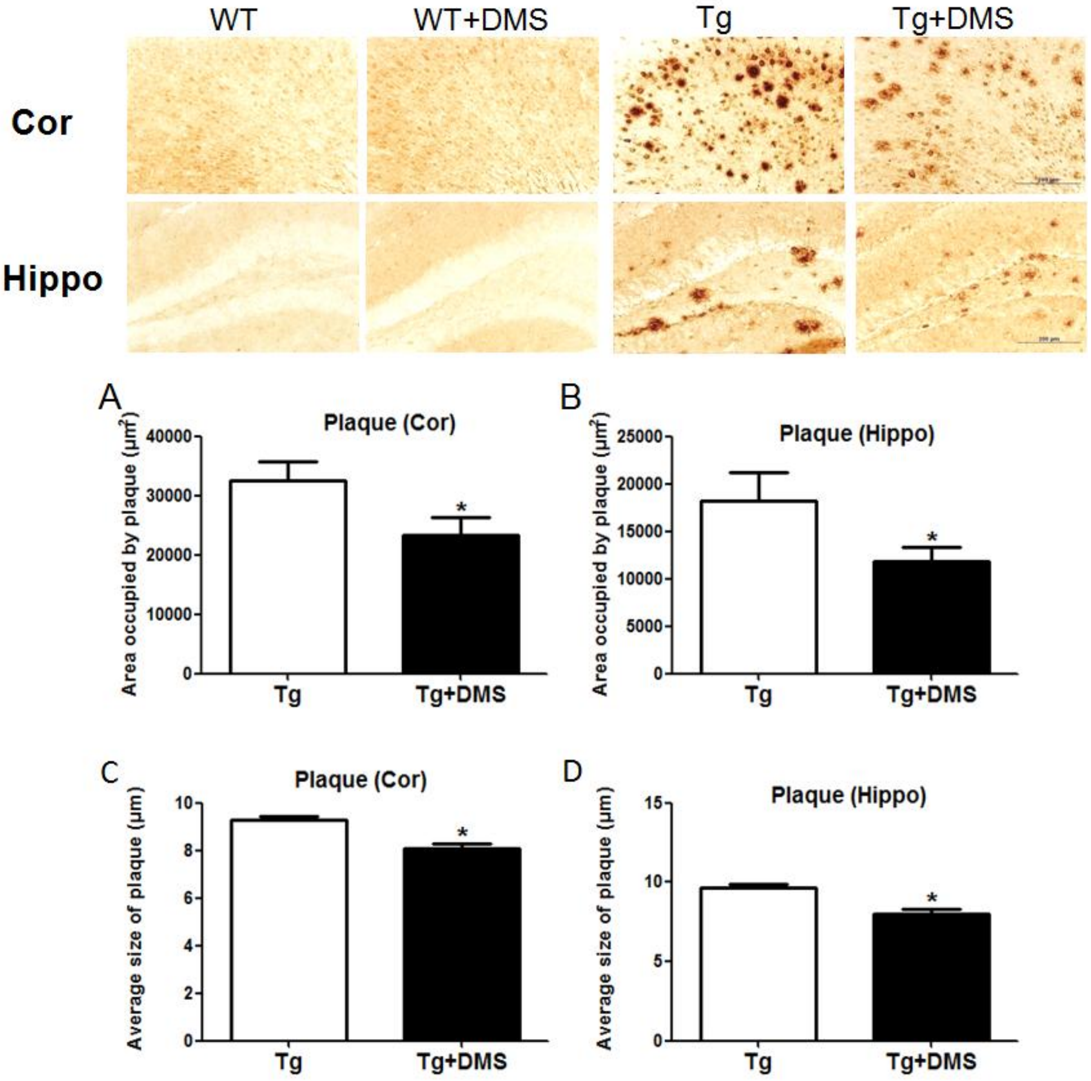

FIGURE S2 | DMS administration reduced the generation of Aβ in the cortex and hippocampus of 5XFAD mice. Senile plaques were detected by immunohistochemical staining with Aβ-specific antibody, 6E10. Massive amyloid plaques were predominantly distributed on the cortex and hippocampus of the 5XFAD mice, whearas no detectable positive signal in WT brain was found. Compared to the Tg group, DMS treatment effectively reduced the area occupied by Aβ positive plaques **(A, B)** and the average size of plaques **(C, D)** in the cortex (Cor) and hippocampus (Hippo), respectively. All values were represented as mean ± SEM, n = 6/group. *p<0.05 vs. Tg, Student's t-test. Scale bar = 200 μm.

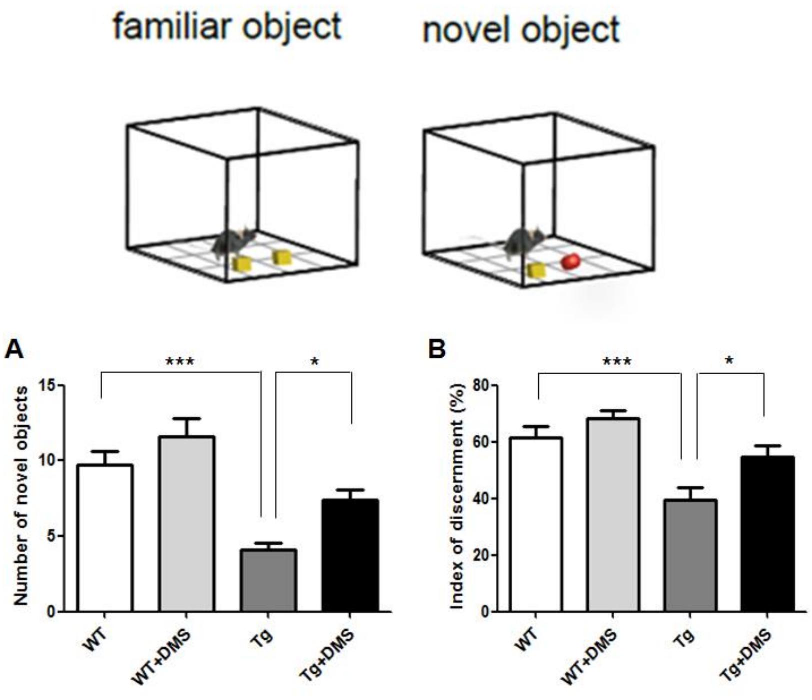

FIGURE S3 | DMS treatment improved the recognition capibility for novel object in 5XFAD mice. Compared to WT mice, 5XFAD mice showed decreased times **(A)** and time **(B)** to interact with novel object, while DMS treatment increased the trend to interact with novel object of 5XFAD. All values were represented as mean ± SEM, n =11/group. *p<0.05, ***p<0.001 vs. Tg, ANOVA.
